# Inhibitory Potential of Cannabis Biomass Extracts on Livestock-Associated Staphylococcal and Streptococcal Pathogens

**DOI:** 10.3390/microorganisms13020432

**Published:** 2025-02-17

**Authors:** Tereza Paulová, Lucie Malíková, Davide Lanzoni, Tomáš Taubner, Matěj Malík, Markéta Houdková, Eva Pěchoučková

**Affiliations:** 1 Department of Microbiology, Nutrition and Dietetics, Faculty of Agrobiology, Food and Natural Resources, Czech University of Life Sciences Prague, Kamýcká 129, 165 21 Prague, Czech Republic; paulovat@af.czu.cz (T.P.); malikovalucie@af.czu.cz (L.M.); 2 Department of Nutritional Physiology and Animal Product Quality, Institute of Animal Science Prague (IAS), Přátelství 815, 104 00 Prague, Czech Republic; taubner.tomas@vuzv.cz; 3 Department of Veterinary Medicine and Animal Sciences (DIVAS), Università Degli Studi di Milano, Via Dell’Università 6, 29600 Lodi, Italy; davide.lanzoni@unimi.it; 4 Department of Agroenviromental Chemistry and Plant Nutrition, Faculty of Agrobiology, Food and Natural Resources, Czech University of Life Sciences Prague, Kamýcká 129, 165 21 Prague, Czech Republic; malikmatej@af.czu.cz; 5 Department of Crop Science and Agroforestry, Faculty of Tropical AgriSciences, Czech University of Life Sciences Prague, Kamýcká 129, 165 21 Prague, Czech Republic; houdkovam@ftz.czu.cz

**Keywords:** *Cannabis sativa* L., animal, antibacterial, inhibition, *Staphylococcus aureus*, *Streptococcus agalactiae*, *Streptococcus dysgalactiae*

## Abstract

Diseases caused by staphylococci and streptococci are a serious burden on livestock production, causing significant losses. In addition, the associated antibiotic resistance of these pathogens often makes treatment impossible or prolonged. *Cannabis sativa* L. contains many compounds with antibacterial properties and shows great potential as a natural antimicrobial agent for agricultural use against both of these bacterial species. The aim of this study was to compare the in vitro antibacterial activity of ethanol extracts from five cultivars of hemp, namely, Bialobrzeskie, Felina 32, Futura 75, mixed and Santhica 27, against *Staphylococcus aureus*, *Streptococcus agalactiae* and *Streptococcus dysgalactiae*. All five cultivars exhibited a certain degree of inhibitory effect against all the pathogens tested with minimum inhibitory concentrations (MICs) ranging from 128 to 2048 μg/mL. The extract from the Santhica 27 cultivar was the most effective antibacterial agent with the lowest MIC value of 128 μg/mL against *Str. agalactiae* and two clinical isolates of *S. aureus*, followed by Bialobrzeskie and mixed cultivars with the same growth-inhibitory potential against *Str. agalactiae*. The extracts from the Felina 32 and Futura 75 cultivars presented only weak activity with MIC values ranging from 256 to 2048 μg/mL. The extract from the Santhica 27 cultivar appears to be a promising product for future use in the treatment of staphylococcal and streptococcal infections in livestock.

## 1. Introduction

Bacterial infections are a critical challenge in livestock farming, severely limiting productivity and contributing to global food insecurity. The World Organisation for Animal Health estimates that 20% of animal production is lost annually due to disease, resulting in substantial economic losses. In particular, livestock diseases reduce global dairy production by 179.5 billion kilograms and meat production by 80 billion kilograms each year, leading to financial losses of approximately USD 358.4 billion [1]. *Staphylococcus aureus* and streptococcal species, such as *Streptococcus agalactiae* and *Streptococcus dysgalactiae*, are among the most significant pathogens affecting livestock and are especially problematic in the dairy industry [2,3].

These pathogens are leading causes of mastitis, an inflammatory disease of the mammary gland, which is the most prevalent and economically detrimental disease in dairy cattle. Mastitis results in both reduced milk quality and yield, with an estimated annual cost of USD 147 per cow [4,5].

Despite reports indicating a decline in the frequency of bacteremia caused by *Str. agalactiae* and *Str. dysgalactiae*, antibiotic resistance among these pathogens is increasing. Resistance to clindamycin, erythromycin and tetracyclines has become a growing concern [6]. For example, *Str. dysgalactiae* isolates exhibit erythromycin minimum inhibitory concentrations (MICs) ranging from 250 to 1000 μg/mL [7]. Similarly, *S. aureus* isolated from pigs was 100% resistant to tetracycline, 88% resistant to penicillin and 64% resistant to clindamycin in a study involving 220 isolates. In beef cattle, resistance rates were reported to be 63.2% for penicillin and 15.8% for both clindamycin and erythromycin [8].

The increasing prevalence of antimicrobial resistance (AMR) in these pathogens presents a serious threat to livestock health, food production, and economic stability, highlighting the urgent need for improved disease management and sustainable antibiotic use in animal husbandry.

This evidence suggests the need to find alternative methods of treatment. One alternative is the development of new antistaphylococcal and antistreptococcal agents involving the isolation and identification of new bioactive chemicals from plants or natural extracts as innovative therapeutics against microbial resistance [9]. Recently, plants and their bioactive substances have come to the forefront in the field of disease protection [10]. *Cannabis sativa* is one of the most widely studied natural agents [11], and not only supports sustainable agriculture and has high nutritional aspects in animal nutrition [12] but is also used in medicine [13] and various antibacterial investigations. The potential of hemp plants in these areas can be attributed to their high content of phytochemicals such as cannabinoids or terpenes [11].

The ability of hemp extracts, specifically phytocannabinoids, to inhibit the growth and spread of bacteria has been investigated in many studies [14]. However, the antimicrobial effects of phytocannabinoids depend on various factors including the composition of the specific cannabinoids used and their concentrations [14]. Given the strong antibacterial potential of cannabis extracts and the differences in their effects between various cultivars, it is important to understand and explore which variety may be best. Therefore, the aim of this study was to evaluate the differences in the growth inhibition efficacy of ethanol extracts from five different hemp cultivars (Bialobrzeskie, Felina 32, Futura 75, mixed and Santhica 27) on bacteria that cause serious and prevalent livestock diseases, including antibiotic-resistant forms of pathogens such as *S. aureus*, *Str. agalactiae* and *Str. dysgalactiae*. Felina 32 and Futura 75 are approved for cultivation within the European Union, with a cannabidiol content of 2–3% and tetrahydrocannabinol levels well below the legal threshold (below 0.5% for Felina 32 and under 0.12% for Futura 75). The Santhica 27 cultivar is characterised with cannabigerol as the predominant cannabinoid, followed by cannabidiol. In contrast, Bialobrzeskie is characterised by a high fibre content and strong resistance to diseases. To our knowledge, this study is the first to compare the antibacterial effects of ethanol extracts from single hemp cultivars against both antibiotic-resistant and antibiotic-sensitive clinical and standard strains of *S. aureus*, *Str. agalactiae* and *Str. dysgalactiae*.

## 2. Materials and Methods

### 2.1. Materials

Four cultivars of hemp biomass, namely, Bialobrzeskie, Felina 32, Futura 75 and Santhica 27, were obtained from the Czech Hemp Institute (Chraštice, Czech Republic). The Bialobrzeskie, Santhica 27 and Felina 32 varieties were harvested on 10/2021, and the Felina 32 variety was harvested on 9/2022. The mixed variety consisted of Bialobrzeskie, Santhica 27 and Felina 32 at a ratio of 10:10:40.

### 2.2. Methods

#### 2.2.1. Preparation of Plant Extracts

All the dried samples were homogenised and crushed into powder via a friction bowl, and 0.3 g of each sample was added to 10 mL of 96% ethanol (Penta, Praha, Czech Republic). The resulting mixture was vigorously mixed for 60 min on a shaker (IKA KS 130 Basic, Staufen, Germany) (800 motion min^−1^). After extraction, the mixture was filtered under low pressure. The remaining part of the filter was transferred to a flask, and 10 mL of ethanol was added. The same extraction procedure was repeated two times. After three extractions, a total volume of 30 mL was collected in one 50 mL plastic tube (VWR, Stříbrná Skalice, Czech Republic).

#### 2.2.2. Determination of Cannabinoid Contents in the Extracts

For the analysis, 0.5 mL of ethanol extract was filtered through a nylon filter (0.22 µL) (Labicom, Olomouc, Czech Republic), and this volume was diluted 100 times. The solution was transferred to an HPLC vial, and 20 µL was injected into the chromatographic system. Chromatographic analysis was performed according to Czauderna et al. (2024) [15]. Chromatographic separation of 14 cannabinoids (CBDVA—cannabidivarinic acid, CBDV—cannabidivarin, CBDA—cannabidiolic acid, CBGA—cannabigerolic acid, CBG—cannabigerol, CBD—cannabidiol, THCV—tetrahydrocannabidivarin, THCVA—tetrahydrocannabidivarinic acid, CBN—cannabinol, Δ^9^-THC—Δ^9^-tetrahydrocannabinol, Δ^8^-THC—Δ^8^-tetrahydrocannabinol, CBC—cannabichromene, THCA—tetrahydrocannabinolic, CBCA—cannabichromenic acid) was conducted using a NexLeaf guard column (5 × 4.6 mm), Shim-Pak column (Shimadzu 2.2 µm, length of 75 mm and inner diameter of 3 mm) and two Phenomenex C18–columns (Synergi 2.5 µm, Hydro RP, 100 Å, length of 100 mm and inner diameter of 3 mm). A high-performance liquid chromatography system (SHIMADZU, Kyoto, Japan) that incorporated a liquid chromatograph LC-20AD, an autosampler SIL-20AC, a column oven CTO-20AC, a communication bus module CBM-20A and a diode array detector (DAD) SPD-M20A was used in this experiment. The column heater maintained a 40 °C temperature. All samples were analysed by using a linear gradient of 99.9% HPLC-grade purity acetonitrile (Chem-Lab NV, Zedelgem, Belgium) with 0.1% of formic acid (Lachner, Neratovice, Czech Republic) (*v*/*v*; solvent A), HPLC-grade purity water (refined by the ElixTM water purification system, Milipore, Canada) with 0.1% of formic acid (Lachner, Neratovice, Czech Republic) (*v*/*v*; solvent B) and 99.8% HPLC-grade purity methanol (Chem-Lab NV, Zedelgem, Belgium) (*v*/*v*; solvent C). The maximum system pressure was 24 MPa.

#### 2.2.3. Bacterial Strains and Culture Media

In this study, four *S. aureus* strains and two strains of the genus *Streptococcus* were used. The standard strains of *S. aureus* ATCC 29213 and ATCC 43300 were obtained from the American Type Culture Collection (ATCC, Rockville, MD, USA). Clinical isolates (SA1, SA3) were purchased from Motol University Hospital (Prague, Czech Republic) and identified via matrix-assisted laser desorption/ionisation time-of-flight mass spectrometry (MALDI-TOF-MS), as described previously by Rondevaldova et al. (2018) [16]. *Str. agalactiae* DSM 2134 and *Str. dysgalactiae* DSM 20662 were obtained from the German Resource Centre for Biological Material (Braunschweig, Germany). Cation-adjusted Mueller–Hinton Broth (Oxoid, Basingstoke, UK) was used as the cultivation and assay medium for all tested strains of *S. aureus*, while the streptococcal strains were cultured in tryptone soya broth (Oxoid). All the growth media used were equilibrated to pH 7.6 with Trizma base (Sigma-Aldrich, St. Louis, MO, USA).

#### 2.2.4. Antibacterial Assay

The antibacterial potential of cannabis extracts was determined via standard broth microdilution in 96-well microtiter plates following the protocols of the Clinical Laboratory Standards Institute guidelines [17], with slight modifications proposed by Cos et al. (2006) for more effective assessment of the anti-infective potential of natural products [18]. Serial dilutions of cannabis extracts were prepared in an appropriate growth medium (90 µg/mL), ranging from 16 to 2048 µg/mL, via a manual multichannel pipette (Eppendorf, Wesseling Germany). The plates were inoculated with a bacterial suspension at a final density of 5 × 10^5^ CFU/mL via the McFarland Densitometer Biosan DEN 1 (BioTech, Prague, Czech Republic) and incubated at 37 °C for 24 h (Biological Thermostat BT 120 M, Laboratorní přístoje Praha, Prague, Czech Republic) under aerobic conditions. The growth of the *S. aureus* strains was then assessed by turbidity, which was determined with an Infinite 200 PRO microplate reader (Tecan, Männedorf, Switzerland) at 405 nm according to Cos et al. (2006) [18]. The MICs were determined as the lowest concentrations that inhibited bacterial growth by ≥80% compared with that of the agent-free growth control and are expressed in µg/mL. Noninoculated and inoculated wells containing broth were included as sterile and negative (growth) controls. The ethanol used as a negative control did not inhibit any of the strains tested. *S. aureus*, *Str. agalactiae* and *Str. dysgalactiae* were checked for positive effects on the antibiotic controls oxacillin and penicillin. All samples were assayed as three independent experiments each carried out in triplicate and the results are expressed as median/modal MIC values. According to the widely accepted norm in MIC testing, the mode and median were used for the final value calculation when the triplicate endpoints were within the two- and three-dilution ranges, respectively. All tests were performed as three independent experiments, each carried out in triplicate, and the results are presented as modal values.

##### Data Analysis

For the calculation, the values obtained from Tecan were uploaded into Microsoft Excel where the values were calculated and then the results were expressed as median/modal MIC values.

## 3. Results

### 3.1. Minimum Inhibitory Concetration of Cannabis Extracts

The results of the in vitro growth-inhibitory effects of cannabis extracts against bacterial strains of *S. aureus*, *Str. agalactiae* and *Str. dysgalactiae* determined via the broth microdilution method are summarised in Table 1.

The lowest MIC value (128 µg/mL) was recorded for cultivar Santhica 27 against *Str. agalactiae* and both clinical strains (SA1 and SA3) of *S. aureus*, followed by extracts from Bialobrzeskie and mixed cultivars with the same growth-inhibitory potential against *Str. agalactiae*. Slightly higher MIC values (256 µg/mL) were observed for the Santhica 27 cultivar than for the standard strains *S. aureus* (ATCC 29213 and ATCC 43300), followed by the Bialobrzeskie (256 and 512 µg/mL) and mixed (512 µg/mL) cultivars. The same MIC (256 µg/mL) was recorded for the Bialobrzeskie and mixed cultivars against both clinical strains (SA1 and SA3) of *S. aureus* and for the Felina 32 and Futura 75 (MICs of 256 and 512 µg/mL, respectively) cultivars against *Str. agalactiae* and clinical strains (SA1 and SA3) of *S. aureus*. In contrast, cultivars Felina 32 and Futura 75 exhibited only weak inhibitory activity (1024 µg/mL) against the standard strains of *S. aureus*. *Str. dysgalactiae* was found as the most resistant bacterial strain, as the same weak growth-inhibitory activity was observed for all five cultivars (MIC of 2048 µg/mL). The slight differences between our results and previously published data may be explained by the different methodologies and bacterial strains used.

### 3.2. Contents of Cannabinoids in Extracts

**Figure 1:**
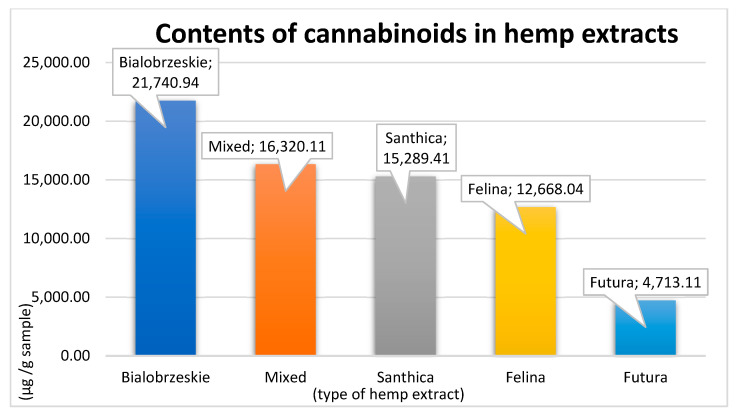
Contents of cannabinoids in hemp extracts.

Differences in cannabinoid contents were observed among the five extracts tested. The total analysed cannabinoid content of each extract is shown in Figure 1. The contents of the specific cannabinoids analysed are shown in Table 2.

The most abundant cannabinoid present in the analysed extracts was CBDA followed by CBD, THCA and CBCA. In contrast, THCV, THCVA and Δ^8^-THC were not detected in the extracts. CBDV, CBN and CBC were recorded at low concentrations.

The highest content of observed cannabinoids was detected in the Bialobrzeskie extract (21,740.94 µg/g sample) followed by the mixed extract (16,320.11 µg/g sample), and the Santhica 27 extract (15,289.41 µg/g sample) had the third highest content. Fewer cannabinoids were detected in the Felina 32 extract (12,668.04 µg/g sample) and the lowest amount of cannabinoids was recorded in the Futura 75 extract (4713.11 µg/g sample).

## 4. Discussion

In general, all of the hemp ethanol extracts showed some degree of in vitro antibacterial activity against all of the bacterial strains tested, including both the antibiotic-resistant and antibiotic-sensitive forms; however, their potency varied substantially from 128 μg/mL to 1024 μg/mL. While these MIC values are higher than those reported in the literature for individual cannabinoids, our results are partially consistent with those obtained in recent studies demonstrating the growth-inhibitory effects of crude cannabis extracts against staphylococcal strains over a wide MIC range from 8 to 8552 µg/mL [19,20,21,22]. Specifically, lower values were reported by Skala et al. (2022), who reported an MIC of 8 μg/mL for an ethanolic cannabis extract against *S. aureus* (ATCC 29213) [22]. Additionally, the MICs for the seed (25 μg/mL) and whole plant (50 μg/mL) for the ethanol extracts were measured via the microdilution broth method [19]. Although the MIC value reported by Ali et al. (2012) is significantly lower than that from our findings, it cannot be fully compared with our findings because the cannabinoid content of the plant was not observed [2]. In contrast, in the study by Skala et al. (2022) [22], the total cannabinoid content of the two ethanol extracts studied was higher (245,940 and 329,100 µg/g) than that in our study (Figure 1). However, the total content cannot be fully compared due to the different amounts of cannabinoids analysed (7 tested extracts vs. 14 tested extracts in our study). However, the amounts of individual cannabinoids contained in the extracts were greater (80–261,750 µg/g) than those in our study (0–15,208.79 µg/g). In addition, our extracts did not contain as much Δ^9^-THC (40.61–513.27 µg/g), CBC (105.28–301.71 µg/g) or CBG (17.12–1163.19 µg/g) as the extracts described in Skala et al. (2022) [22] did, where the values ranged from 35,240 to 51,230 µg/g); 1350–1450 µg/g and 540–1050 µg/g for Δ^9^-THC, CBC and CBG, respectively; this could explain the lower MICs reported by Skala et al. (2022) [22], as the antibacterial activity of these individual cannabinoids has been well studied [11]. However, the antibacterial activity was determined for whole extracts containing different amounts and contents of cannabinoids and other substances that interact with each other. In addition, Skala et al. (2022) used a medicinal cannabis strain containing relatively high amounts of cannabinoids [22]. Our study used industrial hemp, which generally contains lower amounts of cannabinoids but is permitted for use in livestock under legislation (0.2% THC content [23]), and, thus, could be used in this area of research in subsequent in vivo experiments. Giselle et al. (2023) reported values comparable to our findings for *S. aureus* (ATCC 29213), in which the MIC of an inflorescence extract was 115.25 μg/mL, and the MIC of a root extract was 8552.38 μg/mL [21]. Additionally, Kaur et al. (2015) determined the MIC of a methanolic extract of *C. sativa* to be 1560 μg/mL via the agar well diffusion method [20].

In streptococci, the antibacterial activity of specific cannabinoids was investigated in strains of *Streptococcus mutans* (MIC 2.5 μg/mL), *Streptococcus sanguis* (MIC 1 μg/mL), *Streptococcus sobrinus* (MIC 5 μg/mL), *Streptococcus salivarius* (MIC 5 μg/mL) [24], *Streptococcus pyogenes* (2–5 μg/mL), *Streptococcus milleri* (1–2 μg/mL) [25] and *Streptococcus pneumoniae* (1–2 μg/mL) [26]. The values reported in this study are greater than those reported in the literature, which has focused on assessing the activity of individual cannabinoids present in *C. sativa*, probably because samples from whole *C. sativa* extracts may be expected to display interactions among the substances present or different mechanisms of action. The MIC values in our study are similar to those of ethanol extracts investigated against *Str. pyogenes* (128 μg/mL) by Skala et al. (2022) [22].

The cannabinoid content of *C. sativa* depends on many biotic and abiotic factors such as nutrient availability, growth cycle stage, storage, light intensity and ambient temperature [27]. Differences in the cannabinoid content were observed among the five extracts tested, which may influence their antibacterial activity; this is also indicated by our results, in which higher antibacterial activity was observed in extracts with higher cannabinoid contents (Bialobrzeskie, mixed and Santhica 27) and lower antibacterial activity was observed in extracts with lower cannabinoid contents (Felina 32 and Futura 75). These results partially correlate with the different antibacterial activities of the flower and root extracts. The antibacterial activity of cannabinoids in flowers has been demonstrated at lower MICs (1–16 μg/mL) [14] and (MIC 115.25 μg/mL). Meanwhile, in the root extracts, a relatively high MIC was detected, and at the same time, the absence of cannabinoids was noted (MIC 8552.38 μg/mL) [21]. These findings suggest that the cannabinoid content could affect the antibacterial activity of cannabis. However, it should be noted that the antibacterial activity cannot be attributed solely to the cannabinoids themselves but rather to the synergy of substances contained in cannabis in general [21].

To our knowledge, this study is the first to compare the antibacterial effects of ethanol extracts from single hemp cultivars against both antibiotic-resistant and antibiotic-sensitive clinical and standard *S. aureus*, *Str. agalactiae* and *Str. dysgalactiae* strains.

## 5. Conclusions

In this study, the antibacterial activity of ethanol extracts obtained from five different hemp cultivars (Felina 32, Futura 75, mixed, Santhica 27 and Bialobrzeskie) against various strains of *S. aureus*, *Str. agalactiae* and *Str. dysgalactiae*, including their antibiotic-resistant and antibiotic-sensitive forms, was investigated. All five ethanol cannabis extracts possessed specific growth-inhibitory potential against the bacteria tested, while Santhica 27 was identified as the most effective cultivar followed by the mixed and Bialobrzeskie cultivars. In the case of the Felina 32 and Futura 75 cultivars, only weak antibacterial activity was observed. In the context of ever-increasing bacterial resistance to antibiotics, it is necessary to find alternatives to antibiotic treatment, and cannabis has great potential in this area. However, the composition of cannabis is very diverse, which was demonstrated by our findings that illuminated the different inhibitory activities of varying cannabis extracts against different bacteria within different cultivars. Within the scope of this study, the Santhica 27, Bialobrzeskie and mixed cultivars appeared to be the most promising for future use in terms of their antibacterial activity. Therefore, further investigations on the effects of the composition of cannabis extracts on antibacterial activity would be useful in future research. Nevertheless, further research focused on toxicology and in vivo determination will be necessary to verify the potential of cannabis for therapeutic applications in staphylococcal and streptococcal infections.

## Figures and Tables

**Table 1:** Antibacterial activity of ethanol extracts from different cultivars of whole plants of *C. sativa* against strains of *Staphylococcus aureus*, *Streptococcus agalactiae* and *Streptococcus dysgalactiae*.

Bacterium	Strain	Cultivars of Cannabis Sativa/MIC (µg/mL)				
		Bialobrzeskie	Felina 32	Futura 75	Mixed	Santhica 27
*S. aureus*	ATCC 29213	512	1024	1024	512	256
	ATCC 43300	256	1024	1024	512	256
	SA1	256	512	512	256	128
	SA3	256	512	512	256	128
*Str. agalactiae*	DSM 2134	128	256	256	128	128
*Str. dysgalactiae*	DSM 20662	1024	2048	2048	1024	1024

Abbreviations: *S. aureus*—*Staphylococcus aureus*; ATCC—American Type Culture Collection; SA1, SA3—clinical isolates of *Staphylococcus aureus*; *Str. agalactiae*—*Streptococcus agalactiae*; *Str. dysgalactiae*—*Streptococcus dysgalactiae*; DSM—German Resource Centre for Biological Material; MIC—minimum inhibitory concentration (expressed as an average of three independent experiments performed in triplicate).

**Table 2:** Contents of cannabinoids in different cultivars of whole plants of *C. sativa*.

Observed Cannabinoids	Contents of Cannabinoids in Extracts (µg/g Sample)				
	Bialobrzeskie	Felina 32	Futura 75	Mixed	Santhica 27
CBDVA	360.58	144.94	63.82	258.58	ND
CBDV	ND	8.65	14.90	ND	ND
CBDA	15,208.79	7404.60	2776.69	10,206.44	2650.84
CBGA	471.21	2727.43	213.81	2215.25	10,450.78
CBG	89.78	269.63	17.12	232.44	1163.19
CBD	2839.14	521.72	1257.34	1590.11	370.69
THCV	ND	ND	ND	ND	ND
THCVA	ND	ND	ND	ND	ND
CBN	ND	ND	15.60	ND	ND
Δ^9^-THC	513.27	ND	40.61	261.37	59.36
Δ^8^-THC	ND	ND	ND	ND	ND
CBC	ND	301.71	105.28	ND	ND
THCA	1350.45	314.58	53.32	746.26	194.74
CBCA	907.73	974.79	154.62	809.65	399.81
Total value	21,740.94	12,668.04	4713.11	16,320.11	15,289.41

Abbreviations: CBDVA—cannabidivarinic acid, CBDV—cannabidivarin, CBDA—cannabidiolic acid, CBGA—cannabigerolic acid, CBG—cannabigerol, CBD—cannabidiol, THCV—tetrahydrocannabidivarin, THCVA—tetrahydrocannabidivarinic acid, CBN—cannabinol, Δ^9^-THC—Δ^9^-tetrahydrocannabinol, Δ^8^-THC—Δ^8^-tetrahydrocannabinol, CBC—cannabichromene, THCA—tetrahydrocannabinolic, CBCA—cannabichromenic acid, ND—not detected.

## Data Availability

The original contributions presented in the study are included in the article, further inquiries can be directed to the corresponding author.

## References

[1] HealthforAnimals (2023). Animal Health and Sustainability: A Global Data Analysis.

[2] Mesquita A.A., Rocha C.M.B.M., Bruhn F.R.P., Custódio D.A.C., Braz M.S., Pinto S.M., Silva D.B., Costa G.M. (2019). *Staphylococcus aureus* and *Streptococcus agalactiae*: Prevalence, resistance to antimicrobials, and their relationship with the milk quality of dairy cattle herds in Minas Gerais state, Brazil. Pesqui. Vet. Bras..

[3] Taponen S., Liski E., Heikkilä A.M., Pyörälä S. (2017). Factors associated with intramammary infection in dairy cows caused by coagulase-negative staphylococci, *Staphylococcus aureus*, *Streptococcus uberis*, *Streptococcus dysgalactiae*, *Corynebacterium bovis*, or *Escherichia coli*. J. Dairy Sci..

[4] Saro J., Stádník L., Bláhová P., Huguet S., Brožová H., Ducháček J. (2024). A decision support system based on disease scoring enables dairy farmers to proactively improve herd health. Czech J. Anim. Sci..

[5] Cheng W.N., Han S.G. (2020). Bovine mastitis: Risk factors, therapeutic strategies, and alternative treatments—A review. Asian-Australas. J. Anim. Sci..

[6] Guy R., Coelho J., Blakey E., Broughton K., Lamagni T. (2023). Laboratory Surveillance of Pyogenic and Non-Pyogenic Streptococcal Bacteraemia in England (2022).

[7] Alves-Barroco C., Roma-Rodrigues C., Raposo L.R., Brás C., Diniz M., Caço J., Costa P.M., Santos-Sanches I., Fernandes A.R. (2019). *Streptococcus dysgalactiae* subsp. dysgalactiae isolated from milk of the bovine udder as emerging pathogens: In vitro and in vivo infection of human cells and zebrafish as biological models. MicrobiologyOpen.

[8] Rao S., Linke L., Magnuson R., Jauch L., Hyatt D.R. (2022). Antimicrobial resistance and genetic diversity of *Staphylococcus aureus* collected from livestock, poultry and humans. One Health.

[9] Vaou N., Stavropoulou E., Voidarou C., Tsigalou C., Bezirtzoglou E. (2021). Towards Advances in Medicinal Plant Antimicrobial Activity: A Review Study on Challenges and Future Perspectives. Microorganisms.

[10] Cakir M., Karatas T., Yildirim S. (2024). Protective effects of green tea (*Camellia sinensis*) extract against cypermethrin-induced neurotoxicity in rainbow trout (*Oncorhynchus mykiss*) brain tissues. Czech J. Anim. Sci..

[11] Sionov R.V., Steinberg D. (2022). Anti-Microbial Activity of Phytocannabinoids and Endocannabinoids in the Light of Their Physiological and Pathophysiological Roles. Biomedicines.

[12] Lanzoni D., Skrivanova E., Pinotti L., Rebucci R., Baldi A., Giromini C. (2024). Review: Nutritional aspects of hemp-based products and their effects on health and performance of monogastric animals. Animal.

[13] Bonini S.A., Premoli M., Tambaro S., Kumar A., Maccarinelli G., Memo M., Mastinu A. (2018). *Cannabis sativa*: A comprehensive ethnopharmacological review of a medicinal plant with a long history. J. Ethnopharmacol..

[14] Alfei S., Schito G.C., Schito A.M. (2023). Synthetic Pathways to Non-Psychotropic Phytocannabinoids as Promising Molecules to Develop Novel Antibiotics: A Review. Pharmaceutics.

[15] Czauderna M., Taubner T., Wojtak W. (2024). Comparative Study of Gas and Liquid Chromatography Methods for the Determination of Underivatised Neutral and Acidic Cannabinoids and Cholesterol. Molecules.

[16] Rondevaldova J., Hummelova J., Tauchen J., Kokoska L. (2018). In Vitro Antistaphylococcal Synergistic Effect of Isoflavone Metabolite Demethyltexasin with Amoxicillin and Oxacillin. Microb. Drug Resist..

[17] (2018). Methods for Dilution Antimicrobial Susceptibility Tests for Bacteria That Grow Aerobically.

[18] Cos P., Vlietinck A.J., Berghe D.V., Maes L. (2006). Anti-infective potential of natural products: How to develop a stronger in vitro ‘proof-of-concept’. J. Ethnopharmacol..

[19] Ali E.M.M., Almagboul A.Z.I., Khogali S.M.E., Gergeir U.M.A. (2012). Antimicrobial activity of *Cannabis sativa* L.. Chin. Med..

[20] Kaur S., Sharma C., Chaudhry S., Aman R. (2015). Antimicrobial Potential of Three Common Weeds of Kurukshetra: An in vitro Study. Res. J. Microbiol..

[21] Giselle F., Azucena I., Dalila O., Florencia F., Facundo R., Giulia M., Sandra F., Maggi M., Ramirez C.L. (2023). Antibacterial activity of cannabis (*Cannabis sativa* L.) female inflorescence and root extract against *Paenibacillus larvae*, causal agent of American foulbrood. Biocatal. Agric. Biotechnol..

[22] Skala T., Kahánková Z., Tauchen J., Janatová A., Klouček P., Hubka V., Fraňková A. (2022). Medical cannabis dimethyl ether, ethanol and butane extracts inhibit the in vitro growth of bacteria and dermatophytes causing common skin diseases. Front. Microbiol..

[23] EFSA Panel on Additives and Products or Substances used in Animal Feed (2011). Scientific Opinion on the safety of hemp (*Cannabis genus*) for use as animal feed (FEEDAP). EFSA J..

[24] Aqawi M., Sionov R.V., Gallily R., Friedman M., Steinberg D. (2021). Anti-Bacterial Properties of Cannabigerol Toward Streptococcus mutans. Front. Microbiol..

[25] van Klingeren B., ten Ham M. (1976). Antibacterial activity of Δ^9^-tetrahydrocannabinol and cannabidiol. Antonie van Leeuwenhoek.

[26] Blaskovich M.A.T., Kavanagh A.M., Elliott A.G., Zhang B., Ramu S., Amado M., Lowe G.J., Hinton A.O., Pham D.M.T., Zuegg J. (2021). The antimicrobial potential of cannabidiol. Commun. Biol..

[27] Glivar T., Eržen J., Kreft S., Zagožen M., Čerenak A., Čeh B., Tavčar Benković E. (2020). Cannabinoid content in industrial hemp (*Cannabis sativa* L.) varieties grown in Slovenia. Ind. Crops Prod..

